# A giant squamous cell carcinoma of the skin of the thoracic wall: a case report and review of the literature

**DOI:** 10.1186/s13256-017-1281-8

**Published:** 2017-05-11

**Authors:** Evangelos P. Misiakos, Vasileia Damaskou, Anna Koumarianou, Alina-Roxani Gouloumi, Paul Patapis, Nick Zavras, Anastasios Machairas

**Affiliations:** 1 3rd Department of Surgery, National and Kapodistrian University of Athens, School of Medicine, Attikon University Hospital, Chaidari, Athens, 12462 Greece; 2 2nd Department of Pathology, National and Kapodistrian University of Athens, School of Medicine, Attikon University Hospital, Chaidari, Athens, 12462 Greece; 3 Hematology-Oncology Unit, Fourth Department of Internal Medicine, National and Kapodistrian University of Athens, School of Medicine, Attikon University Hospital, Chaidari, Athens, 12462 Greece

**Keywords:** Squamous, Carcinoma, Cutaneous, Invasion, Metastasis

## Abstract

**Background:**

We report a case of a 48-year-old white woman who presented with a huge cutaneous protruding tumor of the thoracic wall below her left breast.

**Case presentation:**

The lesion was excised with clear margins from the adjacent skin, and subcutaneous tissue was left to heal with second intention. A histological examination of the surgical specimen revealed a well-differentiated infiltrative cutaneous squamous cell carcinoma. Our patient neglected to attend our Oncological Department to receive chemotherapy. Today, 12 months after surgery, she is alive and without evidence of disease recurrence.

**Conclusions:**

Cutaneous squamous cell carcinoma can reach a huge size if left untreated. Surgery is the primary mode of treatment, followed by chemotherapy if applicable.

## Background

Squamous cell carcinoma (SCC) of the skin is the second most common type of skin cancer and is steadily increasing in frequency [1, 2]. Although most cutaneous SCCs are diagnosed early and successfully treated, in a small percentage of cases, especially if neglected, they may obtain uncontrollable growth and substantial disfigurement. These cases with giant cutaneous SCCs (maximum diameter >5 cm) can be very difficult to treat and despite aggressive excision can present with recurrence and/or metastases.

Here we present a case with a huge cutaneous tumor on the thoracic wall below the left breast that was excised with optimal clinical result.

## Case presentation

**Fig. 1:**
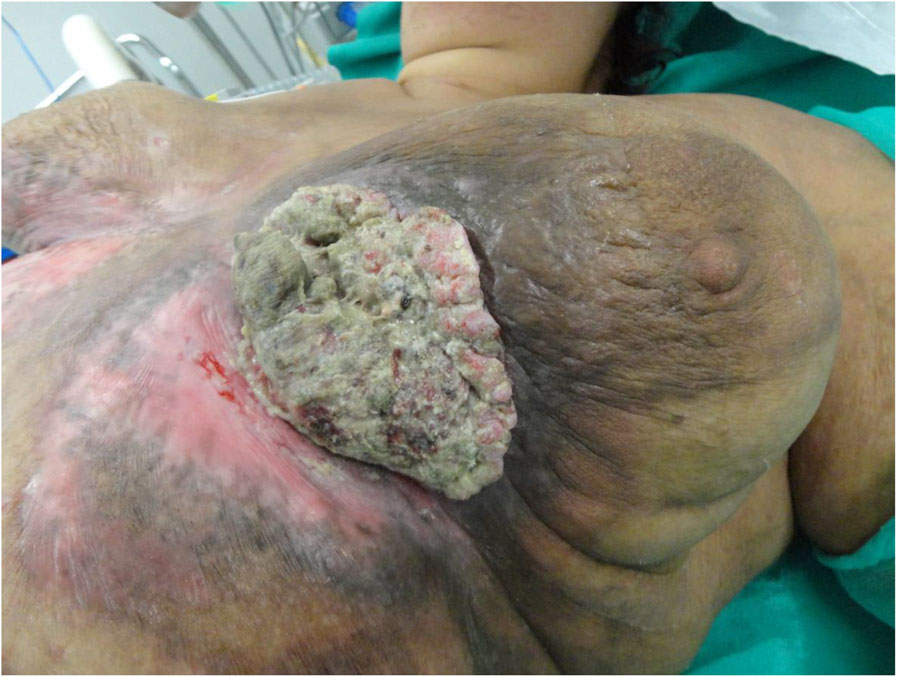
A huge exophytic tumor is prominent on the left thoracic wall, under the left breast. The adjacent skin shows erythematous atrophic areas and extensive hyperpigmentation

**Fig. 2:**
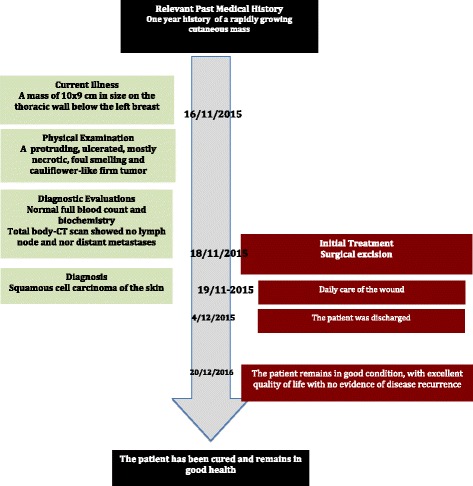
Timeline

A 48-year-old white woman presented with a 1-year history of a rapidly growing cutaneous mass on her thoracic wall below her left breast. The mass was a protruding ulcerated, mostly necrotic, foul smelling, cauliflower-like firm tumor, 10 × 9 cm in size that had developed over a large erythematous skin area (Fig. 1). She reported that the tumor had appeared and reached that size within a 3-month period, however, evidently it was neglected for much longer. Her past medical history included a total thyroidectomy due to goiter, 3 years earlier, psoriasis for 10 years, and schizotypal personality disorder for which she took her medication intermittently, due to poor compliance and social support (Fig. 2).

A full blood count and biochemistry were normal and a total body computed tomography (CT) scan of her thoracic wall showed that the tumor had not infiltrated deep into the musculoskeletal layers of her thoracic wall.

**Fig. 3:**
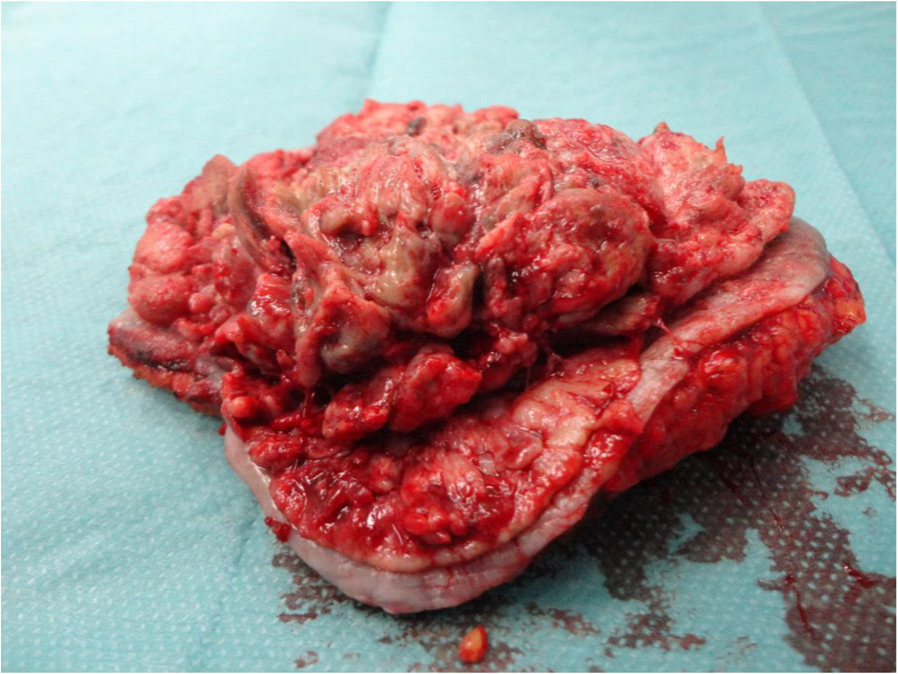
The resected tumor surrounded by a rim of normal skin including the adjacent subcutaneous tissue is shown

No lymph node or distant metastases were noted. She consented to an operation and following the induction of general anesthesia, the tumor was totally excised with a 2 to 3 cm clear margin around it. The tumor seemed to infiltrate the subcutaneous tissue and a 4 cm margin of subcutaneous tissue was excised with the tumor (Fig. 3). Following this wide tumoral excision the surrounding skin could not be approximated and was left to heal slowly with reepithelialization.

After surgery her condition significantly improved. The wound was taken care of daily and she was discharged home after 2 weeks. The wound healed gradually within a couple of months.

**Fig. 4:**
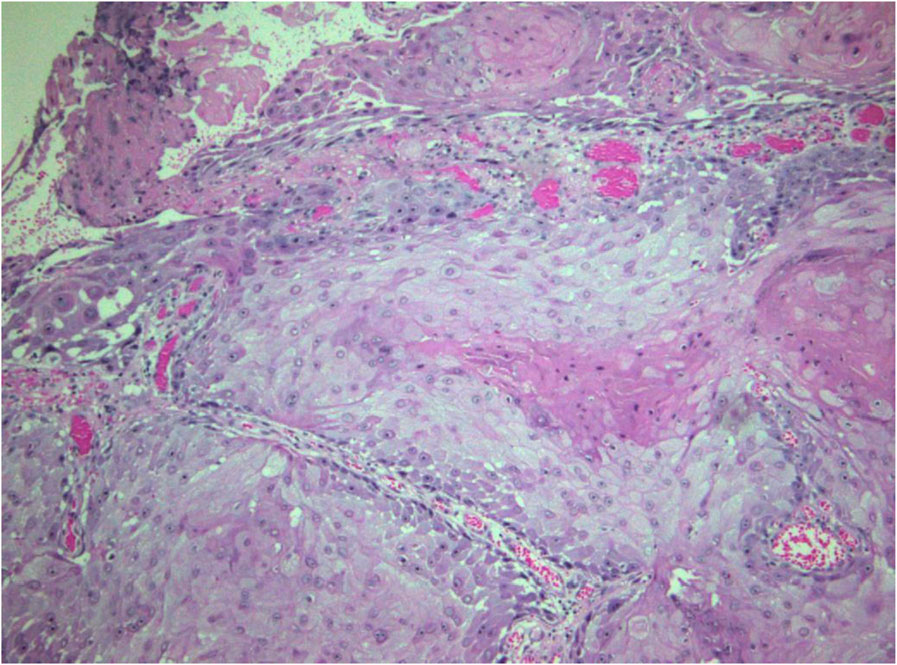
On microscopic examination, a well-differentiated invasive squamous cell carcinoma composed of cells that resemble those of the normal squamous epithelium is shown. Keratinization is evident. Hematoxylin-eosin × 20

A histological examination of the surgical specimen revealed a well-differentiated infiltrative cutaneous SCC. The sections showed a keratinizing (well-differentiated) SCC (Fig. 4) infiltrating deep into underlying subcutaneous tissue with a maximum thickness of 14 mm. Lymphovascular or perineural invasion was not a feature. Excision appeared complete in the sections examined (R0).

**Fig. 5:**
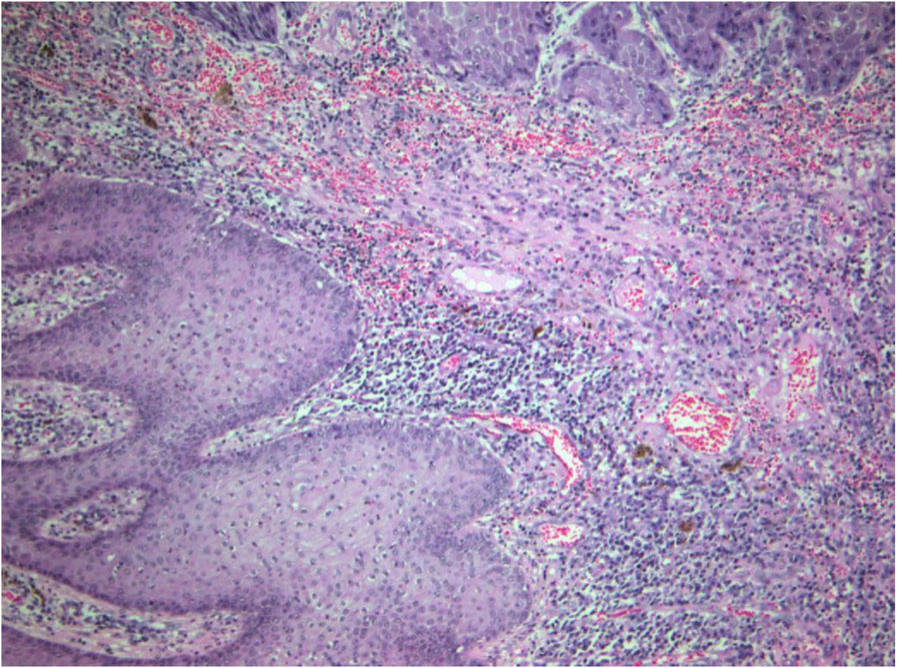
The adjacent skin shows acanthosis and a lichenoid infiltrate with pigment incontinence. Hematoxylin-eosin × 10

The adjacent epidermis was acanthotic with areas of papillomatosis whereas in the dermis a lichenoid lymphocytic infiltrate with pigment incontinence was observed (Fig. 5) suggesting an interface dermatitis. There was no histologic evidence of actinic keratosis or solar elastosis. Staging according to the American Joint Committee on Cancer (seventh edition) was T2N0M0 [3]. Due to the big size of the lesion it was characterized as high risk according to the National Comprehensive Cancer Network (NCCN) criteria [4].

Our patient was advised to go to the Oncological Department of our hospital to receive chemotherapy, which she neglected to do. At present, 12 months after surgery, she remains in good condition, with excellent quality of life and a CT scan showing no evidence of disease recurrence.

## Discussion

Cutaneous SCCs are often associated with radiation exposure, burn scars, varicose ulcers, human papilloma virus infection, chronic inflammation, chronic infection, chemical carcinogens, immunosuppression, and several genetic syndromes [5]. There is also evidence that they may be associated with psoriasis vulgaris; both diseases express bax (bcl-2-associated protein) immunoreactivity [6]. In fact, there have been a few reports of SCCs arising on the grounds of psoriasis vulgaris or lupus vulgaris [7, 8].

The most usual site of giant epithelial malignancies (basal cell carcinoma, SCC) is the scalp [9, 10]. There are, however, cases with other locations, mainly exposed to sun radiation, such as the face and the ear lobes. In any location, they progressively enlarge by peripheral extension or vertical infiltration of the underlying tissue [9]. The most frequent causes of a delay in diagnosis are: low social status, poor personal hygiene, and fear of the diagnosis and the possible consequences [11]. Our patient had all three above causes contributing to the delay in diagnosis and she had a psychiatric disorder. There are not enough data in the literature indicating a possible correlation between neglected tumors and psychiatric disorders, and patient embarrassment seems to be a substantial obstacle, especially in patients with low social status and an underlying psychiatric disorder, as was the case in our patient [11].

Localized SCC of the skin is considered a curable disease. In the case of giant tumors (maximum diameter >5 cm) CT or magnetic resonance imaging (MRI) may be required to accurately assess the extent of the tumor and the possible presence of lymphatic spread [12]. The recommended methods of treatment involve simple excision, cryosurgery, radiation therapy, electrodessication, and curettage [9]. Giant SCC fulfills the criteria of high risk skin carcinomas. Primary treatment is surgical with meticulous examination of the excision margins to ensure R0 resection [10]. For the defect closure, various musculocutaneous flaps and skin transplants have been used. However, the surgical wound can be left open to close gradually with second intention. Surgery is the treatment of choice and the most effective means of achieving cure of any invasive SCC, as it allows confirmation of the tumor type, stage, and examination of the tumor-free status of the resection margins [12]. Complete surgical excision provides very high rates of local control with cure rates of 95% [13]. Other destructive or topical techniques, such as cryotherapy, radiotherapy, curettage, and electrodessication, are reserved for old debilitated patients or for patients who refuse to undergo surgery. After surgery, polychemotherapy is used with cisplatin, 5-fluoruracil, paclitaxel, methotrexate, and other agents with moderate results in cases of advanced inoperable disease [11]. Targeted therapy with epidermal growth factor receptor (EGFR) inhibitors such as cetuximab or erlotinib, are second-line options after monochemotherapy or polychemotherapy failure and disease progression [12].

Follow-up is very important in the management of these tumors, as almost 30 to 50% of patients with SCC are at risk of developing another tumor within 5 years after surgery. Of interest, the majority of these recurrences will develop within 2 years of the initial operation. For that reason, a close follow-up, such as every 6 months, should be applied in patients with resected SCCs, especially for neglected ones with enormous size.

## Conclusions

Localized squamous cell cancer of the skin can reach enormous size if neglected. The invasiveness of the tumor depends on the size, anatomical location, and histological subtype. Surgery is the mainstay of treatment even in giant tumors followed by chemotherapy for the risk of metastatic spread.
